# The Combination of High-Fat Diet and Oral Marijuana Promotes the Development of Fibrosis in the Mouse Corpora Cavernosa

**DOI:** 10.1016/j.esxm.2020.100312

**Published:** 2021-02-01

**Authors:** Sabine Nguyen, Michael Mangubat, Sriram Eleswarapu, Julian B. Wilson, Jocelyn Molina, Andrea Abraham, Jorge N. Artaza, Theodore C. Friedman, Monica G. Ferrini

**Affiliations:** 1 Department of Health and Life Sciences, Charles R. Drew University of Medicine and Science Los Angeles, CA, USA; 2 Department of Internal Medicine, Charles R. Drew University of Medicine and Science Los Angeles, CA, USA; 3 Department of Urology, David Geffen School of Medicine at UCLA, Los Angeles, CA, USA; 4 Department of Medicine, David Geffen School of Medicine at UCLA, Los Angeles, CA, USA

**Keywords:** Cannabis, Marijuana, THC, High-Fat Diet, Fibrosis, Erectile Function

## Abstract

**Introduction:**

The link between cannabis use and erectile dysfunction remains unclear. Moreover, the effect of cannabis in tandem with current Western dietary habits is an area in male sexual health that has yet to be explored. This study seeks to investigate the impact of diet and cannabis on penile health in an animal model.

**Aim:**

To determine the effects of diet and oral cannabis extract on fibrosis and oxidative stress within the corpora cavernosa of mice.

**Methods:**

This is a pilot animal study in which groups of 2-month old C57BL/6J male mice were fed a normal chow diet (NCD) or high-fat diet (HFD) daily and treated with or without either MJ or THC extract for 2 months. After euthanization, mouse penises were isolated and processed for immunohistochemical studies to determine: (i) smooth muscle cell to collagen content, (ii) myofibroblast proliferation, and (iii) anti-oxidative activity.

**Main Outcome Measures:**

Quantitative assessment of immunohistochemical markers of fibrosis and oxidative stress within the corpora cavernosa of mice fed a high-fat diet in combination with either oral marijuana (MJ) or Δ-9-tetrahydrocannabinol extract (THC).

**Results:**

The combination of HFD with MJ resulted in: (i) a decrease in the smooth/collagen ratio in the corpora cavernosa, (ii) an increase in alpha-smooth muscle actin expression in the tunica albuginea compatible with myofibroblast proliferation, and (iii) a decrease in heme oxygenase 1 expression indicating an increase in oxidative stress. Significant histological changes were not observed in the HFD + THC group.

**Conclusions:**

HFD combined with oral MJ extract led to structural alterations in erectile tissue that are associated with accelerated corporal fibrosis. However, the addition of THC to the diet did not exacerbate histological changes within the corpora. Further studies are warranted to elucidate the discrepant effects between MJ and THC in order to optimize the therapeutic potential of cannabis and minimize its adverse effects on penile health.

**S Nguyen, M Mangubat, S Eleswarapu, et al. The Combination of High-Fat Diet and Oral Marijuana Promotes the Development of Fibrosis in the Mouse Corpora Cavernosa. Sex Med 2021;9:100312.**

## Introduction

As the legalization of cannabis continues to be liberalized for medicinal and recreational purposes, its consumption is expected to continue to rise.^1^ As of 2020, cannabis has been legalized in 33 states for medicinal purposes and in 11 states for recreational use.^2^ Yet, its distribution and use remain largely unregulated. According to the 2015 National Survey on Drug Use and Health in the United States, approximately 22.2 million Americans aged 12 and older reported current marijuana use within the past month, with a predominant prevalence of use in males between the ages of 18 and 25 years old. Research on cannabis both as medical therapy and on its adverse health effects is still nascent.^3^^,^^4^

Findings on the effects of cannabis use on male sexual health appear paradoxical. While enhanced sexual arousal and experience have been reported in some cannabis users, habitual cannabis use has been linked to erectile dysfunction (ED).^5^, ^6^, ^7^, ^8^, ^9^, ^10^, ^11^ In one study, chronic cannabis smokers demonstrated penile vasculopathy on veno-occlusive plethysmography.^6^ With studies suggesting potential adverse effects on erectile function, the impact of cannabis on ED requires further investigation.^5^^,^^6^^,^^8^

An explanation for the paradox between the reported enhancement in sexual experience and impairment in erectile function with cannabis use may be attributed to the ubiquity of endocannabinoid receptors, cannabinoid receptor type 1 (CB_1_) and type 2 (CB_2_), throughout the body. Cannabinoids, such as cannabidiol and THC, modulate the activity of dopaminergic and oxytocinergic neurons via brain CB_1_ receptors involved in the regulation of pleasure responses and sexual arousal.^12^^,^^13^ Findings of vasculogenic ED associated with cannabis use may be due to the activation of CB_1_ and CB_2_ receptors in the peripheral vasculature, which has been found to promote atherogenesis and endothelial dysfunction.^14^, ^15^, ^16^

The dried flower and leaves of *Cannabis sativa*, one of the most commonly consumed cannabis strains, contains over 100 pharmacologically active cannabinoids with different potential therapeutic properties and side effects.^17^, ^18^, ^19^, ^20^, ^21^ However, as the cannabinoid compositions in the different strains of cannabis are not standardized, the elicited physiological effects may be unpredictable as well. While purified THC has been studied for clinical use, investigations on marijuana (MJ), or *Cannabis sativa*, plant extracts for medicinal use may be more challenging due to the varying cannabinoid constituents.

Diet can affect risk factors shared by both cardiovascular disease and vasculogenic ED. The Western dietary pattern, which is high in saturated fats and simple carbohydrates, has been associated with chronic systemic inflammation and the development of risk factors such as hypertension, obesity, and dyslipidemia found in vascular disease.^22^, ^23^, ^24^, ^25^, ^26^, ^27^, ^28^, ^29^ Furthermore, in rodent models, a high-fat diet (HFD) has been found to induce changes within the corpora cavernosa suggestive of vasculogenic ED.^30^^,^^31^ These adverse effects may likely be due to the generation of increased reactive oxygen species from the lipid-laden contents in an HFD.^32^, ^33^, ^34^ In a saturated endogenous anti-oxidant system, a redox imbalance can occur, leading to an increase in oxidative stress that results in the deleterious endothelial changes observed in vascular dysfunction.

Although the relationship between cannabis consumption and metabolic conditions such as diabetes and dyslipidemia has not been established, cannabis use has been linked to high caloric intake and cardiovascular dysfunction.^4^^,^^14^^,^^35^, ^36^, ^37^, ^38^, ^39^ Thus, findings that suggest cannabis use and Western diet can negatively impact cardiovascular health may also imply possible harmful effects on erectile function as well. Thus, it is vital to determine whether the combination of diet and cannabis use may have an additive or synergistic effect on erectile tissue health. This study seeks to investigate the effects of HFD and the addition of either MJ or purified THC extract on the erectile tissue of mice. Given that MJ extract contains a mixture of different cannabinoids of uncertain biochemical consequence compared to purified THC, we hypothesize that MJ extract may enhance the inflammatory process brought on by an HFD and lead to observable deleterious changes within the corpora cavernosa.

## Methods

### Animals and Experimental Groups

Two-month old C57BL/6J male mice (Jackson Laboratory, Bar Harbor, ME, USA) were maintained in accordance with a protocol approved by the Charles R. Drew University’s Institutional Animal Care and Use Committee.

Mice were fed either a normal chow diet (NCD) with 5% fat (2.03 kcal/g; Laboratory rodent diet #5001, LabDiet, Richmond, IN, USA) or an HFD containing 60% of calories derived from 26.2% protein, 26.3% carbohydrate, and 34.9% fat (5.24 kcal/g; D12492; Research Diets, New Brunswick, NJ, USA). The HFD was used to simulate the Western dietary pattern. Mice given either diet were untreated or treated with either MJ or THC extract obtained from the National Institute of Drug Abuse drug supply program. We followed a protocol similar to that described by Steffens et al and Varvel et al.^37^^,^^40^ The MJ was an ethanol extraction of *Cannabis sativa* containing 5.23% THC, 0.25% cannabidiol, 0.23% cannabigerol, and 0.23% cannabichromene in dry leaves at 20 mg/kg body weight (BW). Purified THC was dissolved in ethanol at 5 mg/kg BW. Ethanol extracts of MJ and THC were diluted in Boost nutritional drink (240 kcal, 10g protein, 41g carb, 4g fat), which the mice consumed instead of water and in consistent amounts. Control diet mouse groups were given Boost without the cannabis extract. There were six experimental groups (n = 3): (i) Control NCD, (ii) NCD + MJ, (iii) NCD + THC; (iv) Control HFD, (v) HFD + MJ, and (vi) HFD + THC.

After 2 months of exposure, mice were euthanized by CO_2_ inhalation. Mouse penises were isolated, fixed in 10% formalin, and subsequently processed for paraffin-embedded sections.

### Histochemical and Immunohistochemical Study

Five-μm paraffin-embedded cross-sections of mice penile tissue were deparaffinized with xylene, re-hydrated by decreasing concentrations of alcohol, and used for histological and immunohistochemical analysis to determine: a) collagen to smooth muscle cell (SMC) content using Masson’s trichrome staining following the manufacturer’s instructions (Sigma Aldrich, St. Louis, MO, USA), b) myofibroblast expression using a monoclonal antibody against alpha-smooth muscle actin (α-SMA) at 1:400 dilution (Sigma kit, Sigma Diagnostics, St. Louis, MO, USA), and c) anti-oxidative activity using a monoclonal antibody against heme oxygenase 1 (HO-1) at 1:500 dilution (Stressgen, Victoria, BC, Canada). Primary antibodies were omitted in negative controls. Primary antibody immunodetection was achieved using biotinylated secondary antibody (1:200) (Calbiochem, La Jolla, CA, USA) followed by the ABC complex (Vector Labs, Temecula, CA, USA), 0.06% Hydrogen peroxide, and 3-amino-9-ethylcarbazole (AEC) as the chromogen (Sigma, St. Louis, MO, USA) sections were counterstained with hematoxylin.

### Quantitative Image Analysis

For quantitative image analysis, staining intensity was determined by computerized densitometry using the ImagePro Plus 7.1 program (Media Cybernetics, Silver Spring, MD, USA), coupled to Leica microscope with a Leica Camera. For determining the smooth muscle cell/collagen ratio, the total area of stained smooth muscle cells (red) was divided by the total area of stained collagen (blue). The expression of α-SMA and HO-1 was determined by measuring either the % α-SMA or HO-1 positive area over the total corpora cavernosa area. The % α-SMA positive area versus the total sinusoids of the corpora cavernosa was calculated to determine the expression of α-SMA in the sinusoids. In all cases, 4 fields were analyzed per tissue section, with at least 4 matched sections per animal and 3 animals per group.

### Statistical Analysis

Quantitative image analysis (QIA) of histological observations was expressed as mean values ± standard of mean error (SEM). The outcome measures among groups were analyzed by 2-factor ANOVA followed by Tukey post hoc multiple comparisons test using GraphPad V8 (GraphPad Software, La Jolla, CA, USA). Differences were considered statistically significant for *P* ≤ .05.

## Results

### Animal Body Weight

**Figure 1:**
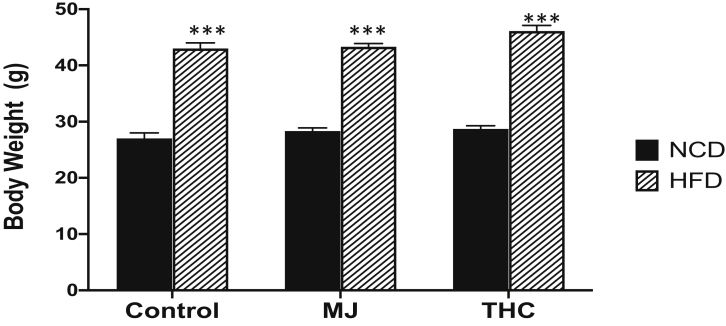
Animal body weights after 2 months of treatment. Control and experimental groups given HFD had a higher average body weight in grams (g) compared to NCD groups over 2 months. Control NCD vs HFD: 27.0 grams (g) ± 1 vs 43g ± 1; NCD + MJ vs HFD + MJ: 28.3 g ± 0.58 vs 43.3 g ± 0.58; HFD + THC vs NCD + THC: 28.7 g ± 0.58 vs 46g ± 1.

After 2 months of treatment, a higher body weight was observed in the HFD animal groups compared to the NCD groups (*P* < .0001). The addition of MJ or THC treatment to diet did not lead to significant body weight changes in the animals compared to their counterparts from the control NCD or HFD only groups (Figure 1).

### HFD and MJ Extract Promotes Structural Changes in the Corpora Cavernosa of mice

**Figure 2:**
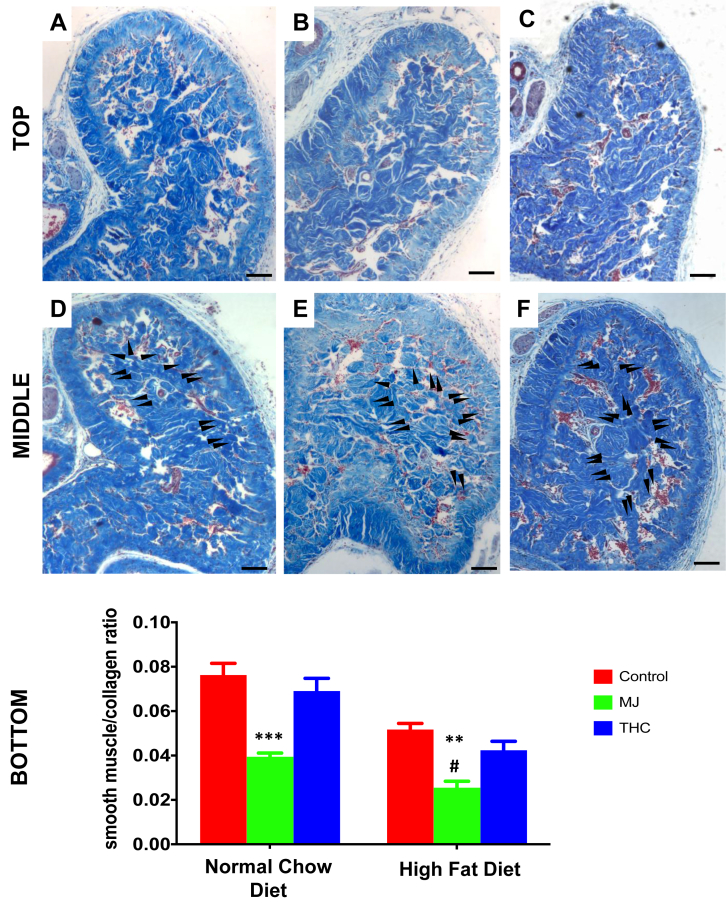
The effect of diet and oral MJ extract on the smooth muscle cell to collagen ratio in mice corpora cavernosa. Cross-section microphotographs of halved mice corpora cavernosa. Top panel: A: Control NCD; B: NCD + MJ; C: NCD + THC; Middle panel: D: Control HFD; E: HFD + MJ; F: HFD + THC. Black arrows point to areas of new collagen deposition (increased areas of blue filling in sinusoids). Bottom panel: Quantitative analysis (QIA) of histological observations expressed as smooth muscle cells(SMCs)/collagen ratio mean ± SEM: NCD + MJ vs NCD (∗∗∗*P* < .001), HFD + MJ vs HFD (∗∗*P* < .01), and HFD + MJ vs NCD + MJ (∗∗#*P* < .05). No statistical significance was computed in NCD + THC and HFD + THC compared to the respective control NCD or HFD groups. Magnification = 100×; 1 bar = 100 μm.

Using Masson’s trichrome staining, analysis of the corpora cavernosa tissue sections revealed a 32% reduction in SMC/collagen ratio in the HFD control mice in comparison to NCD control mice (Figure 2A vs 2C; *P* = .003). The addition of MJ extract to either control NCD or HFD demonstrated an increase in collagen deposition in the sinusoids and further reduced the SMC/collagen ratio within the corpora cavernosa. A 40% reduction in the corpora cavernosa SMC/collagen ratio was observed in the NCD + MJ group compared to the control NCD group (*P* = .001), while a 62% reduction was observed in the HFD + MJ with respect to the control HFD group (*P* = .003) (Top panels: Figure 2A–D; Bottom panel: QIA of the top panels). There was a 38% reduction in SMC/collagen ratio in the corpora cavernosa of the HFD + MJ group compared to the NCD + MJ group (Figure 2B vs 1D; *P* = .015). In contrast, neither NCD + THC nor HFD + THC demonstrated significant changes in the SMC/collagen ratio in comparison to the respective control NCD or HFD groups.

### HFD and MJ Extract Leads to Lipid Vacuole Formation in the Corpora Cavernosa

**Figure 3:**
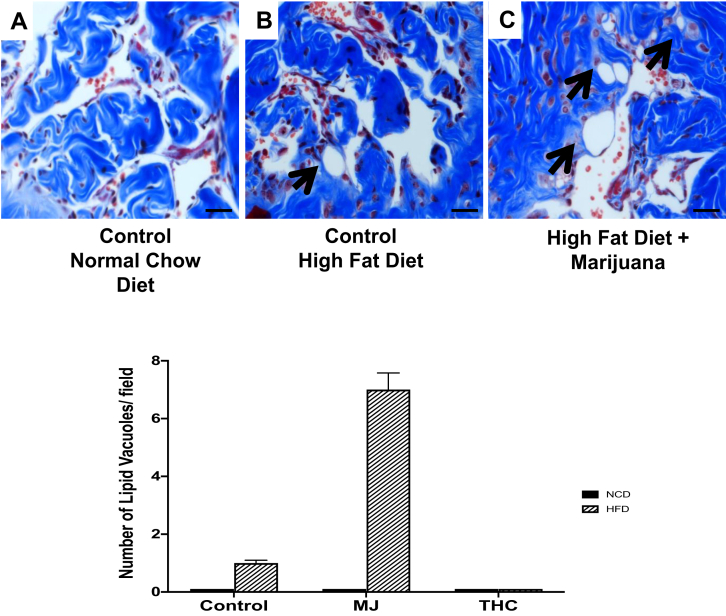
High-fat diet promotes lipid accumulation in the corpora cavernosa. The presence of lipid vacuoles (as indicated by black arrows) are most prominently seen in the corpora cavernosa of mice fed (B) HFD and a combination of (C) HFD + MJ. Fat accumulation was not found in mice administered control NCD or diet with THC combination groups. Magnification: 400×; 1 bar = 50 μm.

Along with changes in SMC and collagen content, lipid vacuoles within the corpora cavernosa were seen primarily in the control HFD group and HFD + MJ groups (Figure 3A–C). The corpora cavernosa of the NCD group (Figure 3A) demonstrated zero lipid vacuoles/40× high power field (hpf), the HFD group (Figure 3B) 1 ± 0.1 lipid vacuole/40× hpf, and HFD + MJ (Figure 3C) 7 ± 0.58 lipid vacuoles/40× hpf. Lipid vacuoles were not found in the cavernosa of the other experimental groups.

### HFD and MJ Extract Promotes Myofibroblast Proliferation in the Tunica Albuginea

**Figure 4:**
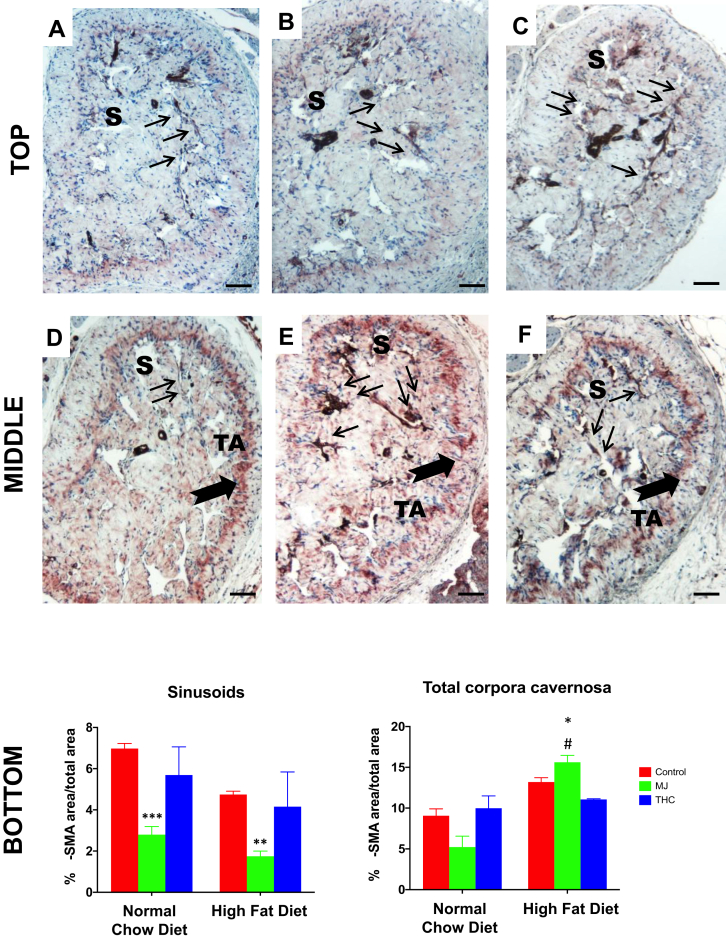
The effect of HFD and oral MJ extract on α-smooth muscle actin expression within the corpora cavernosa. Alpha smooth muscle actin antibody (α-SMA), a surrogate for myofibroblast expression, with AEC as chromogen and counterstaining with hematoxylin. Top panel: A: Control NCD; B: NCD + MJ; C: NCD + THC; Middle panel: D: Control HFD; E: HFD + MJ; F: HFD + THC. Bottom panel: QIA analysis of (α-SMA) determined at the level of the sinusoids and of the total corpora cavernosa. QIA results are expressed as %α-SMA IOD area/total area mean ± SEM: NCD + MJ vs control NCD (∗∗∗*P* < .001), HFD + MJ vs control HFD (∗∗*P* < .01) in the sinusoids; HFD + MJ vs control NCD or HFD (∗*P* < .05) and NCD + MJ vs HFD + MJ (#∗∗*P* < .05) in the tunica albuginea. Localization of α-SMA as demonstrated arrows. Thin arrows indicate the presence of myofibroblasts within the SMCs in the sinusoid (S) area. Thick arrows indicate the presence of myofibroblasts in the tunica albuginea (TA), which is pronounced in HFD groups. THC added to either NCD or HFD did not lead to significant changes in α-SMA expression at the sinusoid or tunica albuginea levels compared to control NCD or HFD. Magnification = 100×; 1 bar = 100 μm.

Alpha-SMA is a marker for both smooth muscle cell and myofibroblast expression. In all NCD groups, as seen in Figure 4A–C, the expression of α-SMA was confined mostly to the sinusoids (S) of the corpora cavernosa. However, in animals given HFD (Figure 4D–F), α-SMA expression was also observed in the tunica albuginea (TA), suggesting an expansion in the transition from fibroblasts into myofibroblasts, which is typically observed in fibrogenesis as a response to inflammation or injury. Thin arrows (S) and thick arrows (TA) in Figure 4 demonstrate the localization of α-SMA within the corpora cavernosa of control NCD (Figure 4A–C) and HFD groups (Figure 4D–F).

For all experimental groups, α-SMA expression was analyzed at the level of the sinusoids and of the total corpora cavernosa (S + TA). At the sinusoid level, where cavernosal SMCs are located, an overall decrease in α-SMA expression was found in the NCD + MJ and HFD + MJ groups compared to the control NCD and HFD groups (Figure 4B vs 4A, *P* = .009; 4E vs 4D, *P* = .0006, respectively; Bottom panel: QIA of top panels). The reduction of α-SMA expression in the sinusoids may be indicative of the reduction in cavernosal SMCs as well.

Interestingly, along with a decrease in the sinusoids, an increase in α-SMA expression was observed in the tunica albuginea and overall total corpora cavernosa (S + TA) in all of the HFD groups compared to the NCD groups (Figure 4D vs 4A, *P* = .031). A 20% increase of α-SMA expression was observed in the HFD + MJ group with respect to the control HFD group (Figure 4E vs 4D, *P* = .013), suggesting that the addition of MJ to HFD exacerbated the fibrotic phenotype. Moreover, the addition of MJ extract to the HFD diet further engendered myofibroblast proliferation, as evidenced by a 3.2-fold increase in total corpora cavernosa α-SMA expression in the HFD + MJ group over the NCD + MJ group (Figure 4E vs 4B; *P* = .0042). The addition of THC to NCD or HFD did not affect α-SMA expression in mice relative to their respective control diets.

### HFD and MJ Extract Reduces Anti-oxidative Activity in the Mouse Corpora Cavernosa

**Figure 5:**
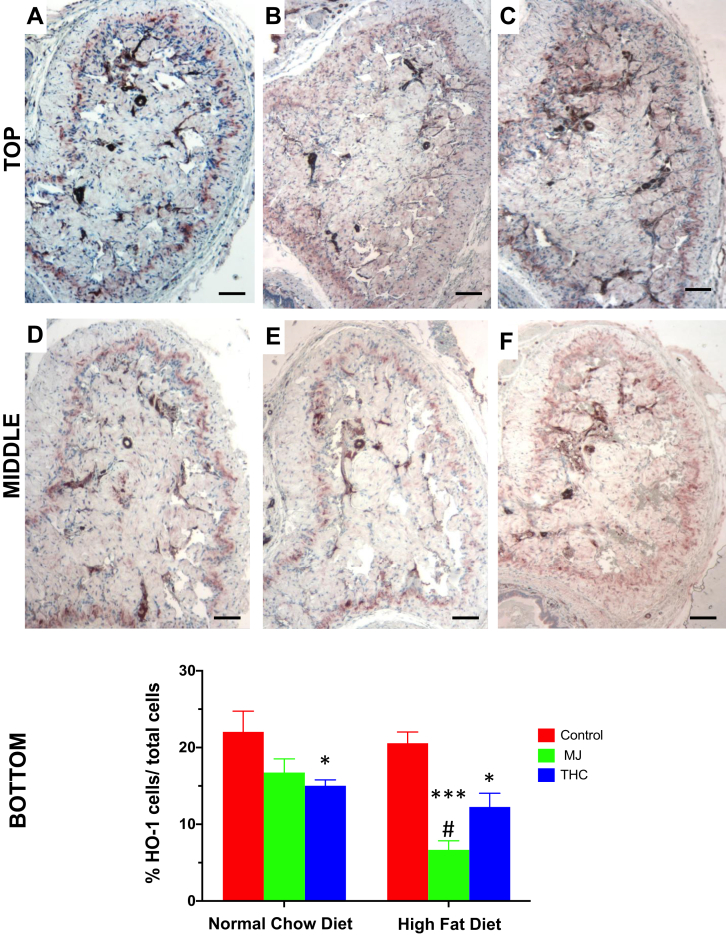
The effect of HFD and oral MJ extract on heme oxygenase 1 expression. Heme oxygenase 1 (HO-1), a marker of antioxidative expression, within the cavernosa was determined by immunohistochemistry. Top panel: A: Control NCD; B: NCD + MJ; C: NCD + THC; Middle panel: D: Control HFD; E: HFD + MJ; F: HFD + THC. Bottom panel: QIA evaluation as %HO-1 cells/total cells means ± SEM. HO-1 expression was significantly reduced in the corpora of HFD + MJ group compared to the control NCD or HFD group (∗∗∗*P* < .001, ∗∗*P* < .01 respectively). A reduction in HO-1 expression was also seen in NCD + THC vs control NCD and HFD + THC vs control HFD (∗*P* < .05). Magnification = 100×; 1 bar = 100 μm.

HO-1, an anti-inflammatory enzyme that combats oxidative stress, was measured to assess for anti-oxidative activity accompanying the histological changes within the corpora cavernosa (Figure 5). There was an approximately 68% decrease in HO-1 expression in the HFD + MJ group compared to HFD control (Figure 5E vs 5D; *P* = .0003). However, no significant effect was seen in the NCD + MJ group compared to NCD control (*P* = .094). A reduction in HO-1 activity was seen in both diet groups with the addition of THC extract. However, this decrease remained relatively stable and was not as pronounced as in the HFD + MJ group. The HFD + THC group demonstrated a 40% decrease in HO-1 activity with respect to HFD control (Figure 5F vs 5D; *P* = .013), while the NCD + THC group revealed a 32% reduction in HO-1 expression compared to NCD control (Figure 5A vs 5C; *P* = .036).

## Discussion

Our results demonstrate that the development of fibrosis appeared most pronounced in the corpora cavernosa of mice given an HFD combined with oral MJ as evidenced by (i) a decrease in SMC to collagen ratio, (ii) an increase in myofibroblast proliferation in the tunica albuginea, and (iii) a reduction in anti-oxidative stress expression. These findings suggest that in the setting of chronic HFD, the addition of MJ consumption may synergistically enhance inflammation and hasten penile fibrosis. In contrast to the effect of MJ extract, the addition of purified THC extract did not appear to exacerbate fibrotic changes. THC may mitigate the inflammatory response, which correlates with the benign impact that is found in the mouse corpora cavernosa of this study.

The histological changes seen within the corpora cavernosa of mice fed HFD + MJ are similar to alterations seen in both vascular atherosclerotic changes and cavernosal veno-occlusive dysfunction.^41^, ^42^, ^43^, ^44^, ^45^ Whether brought on by chronic insults from smoking, diabetes, or obesity in vascular atherosclerosis or the aging process in corporal veno-occlusive dysfunction, a persistent inflammatory state triggers the apoptosis of SMCs and accumulation of collagen. In the penis, these findings are consistent with the progression of fibrosis associated with ED.

Previous investigations alluding that the dysregulation of CB_1_ and CB_2_ receptors can result in atherosclerotic plaque formation in the peripheral vasculature provide insight into the corpora cavernosa histopathology of mice treated with HFD + MJ.^14^^,^^16^^,^^46^^,^^47^ Activation of CB_1_ receptors can lead to the production of lipid-laden macrophages and reactive oxygen species.^15^^,^^47^^,^^48^ While antagonism of CB_2_ receptors found in immunomodulatory cells can dampen the recruitment of proinflammatory cytokines and myofibroblasts, agonism of CB_2_ receptors in the corpora cavernosa may reduce reactive oxygen species.^15^^,^^47^, ^48^, ^49^, ^50^ The cascade of events brought on by CB_1_ and CB_2_ receptor modulation results in the plaque formation seen in vascular endothelium injury, remodeling, and eventual fibrosis. These mechanisms may also be at play in the fibrosis observed in the corpora cavernosa of mice fed HFD + MJ in this study.

The increase in fibroblast to myofibroblast transition in the connective tissue of the tunica albuginea and the accompanying decrease in cavernosal SMC to collagen ratio was significant in the HFD + MJ group. MJ, the extract of *Cannabis sativa* used in this experiment, contains a multitude of natural cannabinoid constituents. This may have led to the non-specific binding of the unidentified constituents to the endocannabinoid receptors, resulting in the activation of the inflammatory cascade and leading to the increased myofibroblast expression. This increase also suggests that MJ may exacerbate the fibrotic progression already underway within the corpora cavernosa oxidative stress already brought on by HFD.

Previous studies using animal models suggest that THC may have anti-inflammatory and anti-oxidative properties, presumably via CB_2_ receptor interaction. THC has been shown to slow the disease progression in rodents induced with rheumatoid arthritis, atherosclerotic plaques, and hepatic fibrosis.^37^^,^^49^, ^50^, ^51^, ^52^, ^53^ In line with these studies, in this experiment, the corpora cavernosa of mice given a diet and THC extract did not demonstrate as a significant reduction in HO-1 expression as in those given a diet and MJ extract. This finding may explain the lack of architectural changes found in the corpora cavernosa of mice administered either NCD + THC or HFD + THC. The reduction in oxidative stress and minimal adverse tissue changes suggest the therapeutic potential of THC as an antioxidant, although more studies are needed. Furthermore, while it appears that purified THC can exert anti-inflammatory and anti-oxidative effects applicable for clinical use, caution may be needed with the use of whole cannabis plant extract in which the cannabinoid constituents are unidentified and unquantified, such as with MJ in this experiment.

As the legalization of cannabis continues to expand for medicinal treatment and recreational use, further delineation of the beneficial and detrimental properties of cannabinoids and their CB_1_ or CB_2_ receptor proclivities is warranted. Additionally, with respect to male sexual health, identifying the presence and localization of endocannabinoid receptors within the corpora cavernosa may further elucidate the effects of cannabis and its components.

There are notable limitations to this study. As this is a pilot study, the authors acknowledge that future experiments will require more mice in each treatment group for an appropriately powered study. Assessments of the corpora cavernosa response to treatments were made based on histological and immunohistochemical changes. While these are adequate surrogates to determine how fibrosis can progress in a structural context, biochemical and physiological data may further corroborate these current findings. Future studies involving gene expression and protein synthesis would be helpful in elucidating mechanisms at play. Erectile function studies with electrical field stimulation and dynamic infusion cavernosometry are a planned next step to identify key structure-function implications to this research.

## Conclusion

HFD, with the addition of oral MJ extract, resulted in a reduction in the smooth muscle cell to collagen content and an increase in myofibroblast proliferation within the corpora cavernosa. These structural alterations in erectile tissue suggest accelerated corporal fibrosis, which may ultimately lead to impaired sexual function. Similar detrimental findings were not readily observed in the diet and THC combination groups. Further studies are warranted to delineate the pharmacological activity of the different cannabinoids in order to optimize the therapeutic potential of cannabis and minimize their adverse health effects.

## Statement of authorship

Sabine Nguyen: Writing - Original Draft, Formal Analysis, Project Administration, Conceptualization, Resources, Writing - Review & Editing; Michael Mangubat: Methodology, Investigation, Resources, Writing - Review & Editing, Funding Acquisition, Writing - Original Draft, Formal Analysis, Project Administration; Sriram Eleswarapu: Writing - Original Draft, Formal Analysis, Project Administration; Julian B. Wilson: Writing - Original Draft, Formal Analysis, Project Administration, Resources, Writing - Review & Editing; Jocelyn Molina: Writing - Original Draft, Formal Analysis, Project Administration, Resources, Writing - Review & Editing; Andrea Abraham: Writing - Original Draft, Formal Analysis, Project Administration; Jorge N. Artaza: Writing - Original Draft, Formal Analysis, Project Administration, Conceptualization, Resources, Writing - Review & Editing, Resources, Writing - Review & Editing; Theodore C. Friedman: Writing - Original Draft, Formal Analysis, Methodology, Investigation, Resources, Writing - Review & Editing, Funding Acquisition, Project Administration, Conceptualization; Monica G. Ferrini: Writing - Original Draft, Formal Analysis, Conceptualization, Methodology, Investigation, Resources, Writing - Review & Editing, Funding Acquisition, Project Administration.
